# Nine Things Genomics Can Tell Us About *Candida auris*

**DOI:** 10.3389/fgene.2020.00351

**Published:** 2020-04-15

**Authors:** Aleksandra D. Chybowska, Delma S. Childers, Rhys A. Farrer

**Affiliations:** ^1^School of Medicine, Medical Sciences, and Nutrition, Institute of Medical Sciences, University of Aberdeen, Aberdeen, United Kingdom; ^2^Aberdeen Fungal Group, Institute of Medical Sciences, University of Aberdeen, Aberdeen, United Kingdom; ^3^Medical Research Council Centre for Medical Mycology at The University of Exeter, Exeter, United Kingdom

**Keywords:** *Candida auris*, genomics, emergence, antifungal resistance mechanisms, virulence factors, epigenetics

## Abstract

*Candida auris* is a recently emerged multidrug-resistant fungal pathogen causing severe illness in hospitalized patients. *C. auris* is most closely related to a few environmental or rarely observed but cosmopolitan *Candida* species. However, *C. auris* is unique in the concern it is generating among public health agencies for its rapid emergence, difficulty to treat, and the likelihood for further and more extensive outbreaks and spread. To date, five geographically distributed and genetically divergent lineages have been identified, none of which includes isolates that were collected prior to 1996. Indeed, *C. auris*’ ecological niche(s) and emergence remain enigmatic, although a number of hypotheses have been proposed. Recent genomic and transcriptomic work has also identified a variety of gene and chromosomal features that may have conferred *C. auris* with several important clinical phenotypes including its drug-resistance and growth at high temperatures. In this review we discuss nine major lines of enquiry into *C. auris* that big-data technologies and analytical approaches are beginning to answer.

## Graphical Abstract

An overview of the nine chapters of this review.

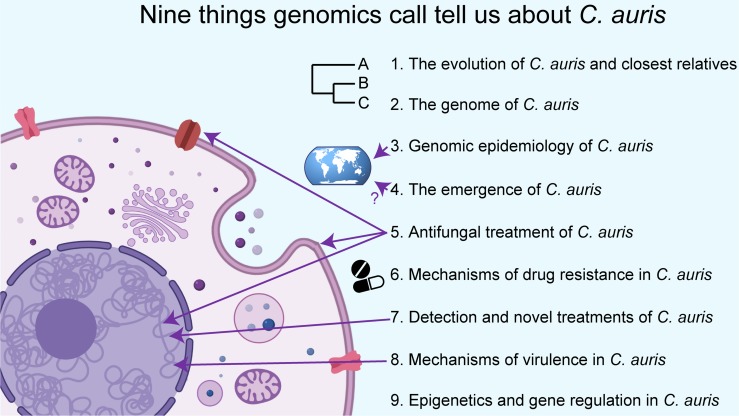

## The Evolution of *C. auris* and Closest Relatives

*Candida auris* was first isolated from the external ear canal (*auris* is Latin for “ear”) of an 70 years old Japanese woman, who was a patient at Tokyo Metropolitan Geriatric Hospital (Tokyo, Japan) in 2009 (Satoh et al., 2009). This first isolate (type strain JCM15448; CBS10913; DSM21092) had several attributes that distinguished it from its closest known relatives, warranting its demarcation as a new species. Unique features of *C. auris* included the ability to grow (albeit slow and with weak growth) at 42°C, while isolates from its three closest relatives (*C. haemulonii*, *C. pseudohaemulonii*, or *C. heveicola*) were unable to grow at this temperature (Satoh et al., 2009). JCM15448 showed a reduced ability to assimilate different carbon sources (galactose, l−sorbose, cellobiose, l−arabinose, ethanol, glycerol, salicin, or citrate), whilst *C. ruelliae* was able to assimilate all of those as carbon sources (although *C. ruelliae* is able to grow at 42°C). JCM15448 produced no pseudohypae while all closest relatives (*C. haemulonii* species complex. *C. heveicola*, and *C. ruelliea*) were able to. The authors concluded that isolate JCM15448 belonged to a new species, and “may have pathogenicity, but this will be elucidated in future studies” (Satoh et al., 2009).

*C. auris* falls within the Clavispora clade of the Metschnikowiaceae family of the order Saccharomycetales, which are ascomycetous (hemiascomycetes) yeasts that reproduce by budding.

Saccharomycetales includes approximately 1000 described species including human commensals and pathogens (e.g., *C. albicans*), plant pathogens (e.g., *Eremothecium gossypii*), species important for baking and brewing (e.g., *Saccharomyces cerevisiae*), species that have associations and interactions with plants and/or arthropods, as well as numerous free living saprobes. The most widely studied *Candida* species are *C. albicans*, *C. tropicalis*, and *C. parapsilosis* from the CTG clade – defined by predominantly translating the codon CTG to serine instead of leucine (Santos et al., 2011). Genetically distinct is *C. glabrata*, which falls in the Nakaseomyces clade (Angoulvant et al., 2016) and is also of growing interest primarily due to its increasing prevalence in nosocomial infections (Toda et al., 2019). These four species together accounted for approximately 90% of species responsible for invasive candidiasis during 2003 (Pfaller and Diekema, 2007). Although *C. auris* is genetically divergent from the other commonly studied CTG clade species, it also translates the codon CTG to serine and is within the CTG clade (Muñoz et al., 2018). *C. auris*’ genetic distance from other more commonly studied species and its recent emergence raises many questions regarding its evolution, epidemiology, and the genetic basis for its pathogenicity and drug resistance.

The phylogenetic relationship of *C. auris* to other known species is still not fully resolved, mainly owing to the rarity of some of its closest relatives. Maximum likelihood phylogenetic reconstruction has revealed that four of the five clades of *C. auris* (with genomic analyses of Clade V not reported yet) are highly genetically related (98.7% average pairwise nucleotide identity), all of which are more distantly related to other *Candida* species that have their genome sequenced including *C. haemulonii*, *C. duobushaemulonii*, and *C. psuedohaemulonii* (88% average pairwise nucleotide identity) (Muñoz et al., 2018). Given the recency of emergence and discovery of already five clades within the *C. auris* species, it seems likely that further clades await discovery. Muñoz et al. found the most closely related species to be *C. haemulonii*, followed by *C. pseudohaemulonii* – both of which are occasionally found in animal or clinical settings (Cendejas-Bueno et al., 2012).

Early non-whole-genome sequencing (specifically 26S rDNA D1/D2 domain) suggested *C. auris* may be more closely related to *C. ruelliae* than *C. haemulonii* (Satoh et al., 2009*)*, a species identified from the flowers of the *Ruellia* species of the Acanthaceae family (Saluja and Prasad, 2008). *C. ruelliae* has not been reported since to the best of our knowledge, and has not had its genome sequenced, thereby hampering efforts to phylogenetically place it, and verify its relationship to *C. auris*. ITS and rDNA sequencing meanwhile suggest that *C. auris* groups phylogenetically with *C. heveicola* (Satoh et al., 2009), a species described by a single isolate YJ2E(T) identified from tree sap from tropical forests in Yunnan and Hainan Provinces in southern China (Wang et al., 2008). Similarly, *C. heveicola* has also not been re-identified or had its full genome sequenced. Evidently, much remains to be known about true genome diversity of both *C. auris* and its closest relatives.

One of the closest known relatives of *C. auris* is *C. haemulonii*, which was first discovered in 1962 (formerly named *Torulopsis haemulonii*) from the gut of a blue-striped grunt fish (*Haemulon scirus*) (van Uden and Kolipinski, 1962), which is found in the Western Atlantic, Gulf of Mexico and the Caribbean. It was also isolated from the skin of dolphins and seawater off the coast of Portugal (van Uden and Kolipinski, 1962; Khan et al., 2007). *C. haemulonii* has also been found from terrestrial sources including the roots of Cassava (*Manihot esculenta*) in Brazil in 2010, and in a laboratory tick colony (*Ornithodoros moubata*) in the Czech Republic in 2001 (Jackson et al., 2019). *C. haemulonii* has also been the cause of candidemia and other bloodstream infections, catheter-related fungemia, osteitis, and even outbreaks in intensive care units (Cendejas-Bueno et al., 2012) including in Kuwait in 2005 (Khan et al., 2007).

In 1993, Lehmann et al. (1993) found *C. haemulonii* to comprise two separate groups (group I and II). In 2012, Cendejas-Bueno et al. (2012) redefined group I as the *C. haemulonii* species, and re-categorized group II as the new species *C. duobushaemulonii* based on genetic differences, ability to grow at different temperatures and on different carbon sources, and drug susceptibilities. *C. duobushaemulonii* has been isolated from patients’ blood and foot ulcers in Asia, Europe, and North America, and has been the cause of recurrent vulvovaginal candidiasis (VVC) in Brazil (Jackson et al., 2019). *C. duobushaemulonii* has also been isolated from a firebug (*Pyrrhocoris apterus*) in Germany (Jackson et al., 2019). In 2006, Sugita et al. (2006) described a closely related but distinct species to *C. haemulonii*, *C. pseudohaemulonii*, which was isolated from the blood of a Thai patient. Recent genome sequencing showed 92% nucleotide genome identity between *C. pseudohaemulonii* and *C. duobushaemulonii* and several chromosomal rearrangements confirming their genetic divergence (Muñoz et al., 2018).

The evolution and phylogenetics of *C. auris* remain enigmatic, due to the disparate ecological sources of its closest relatives, their genetic distance, and that most have not yet had their genomes sequenced and compared, thus precluding detailed population genetic analysis within these species. The discovery of more closely related species by concerted sampling efforts, along with genome sequencing of others already identified but without reference genomes such as *C. ruelliae* or *C. heveicola* may provide additional clues about their and *C. auris*’ evolution.

## The Genome of *C. auris*

To date, five genetically diverse clades have been discovered. The first genome assemblies came from isolates belonging to Clade I, such as the Illumina-based genome of isolate Ci6684 (Chatterjee et al., 2015), and Illumina and Nanopore based genomes of five isolates from an outbreak in London, United Kingdom (Rhodes et al., 2018b). Shortly after, Muñoz et al. (2018) assembled and compared genomes across four (Clade I- > IV) of the then known lineages. Muñoz mapped the genomes into seven chromosomes using optical maps, and recent work using long-reads and telomere-to-telomere assemblies confirm the chromosome count. Genome assembly sizes ranged from 12.1 and 12.7 Mb, and the number of predicted protein coding genes ranged from 5,288 and 5,601, similar to other *Candida* species. The correlation between the genome assembly size and the number of predicted genes was surprisingly negative (*n* = 7, *r*^2^ = 0.0725) (Muñoz et al., 2018). To date, the genome of clade V has not been reported.

The seven chromosomes of *C. auris* have undergone chromosomal rearrangements between clades. For example, inversions and translocations of hundreds of kilobase genomic regions have been reported between Clade I (isolate B8441) and Clade III (isolate B11221) (Muñoz et al., 2018). More recent work using long-read telomere-to-telomere sequencing has shown the Clade II genome appears highly rearranged compared with the other 3 clades, with 2 inversions and 9 translocations resulting in a substantially different karyotype (Muñoz et al., 2019). It was hypothesized that these rearrangements could potentially prevent genetic exchange between those lineages (Muñoz et al., 2018). However, a phylogenetically conserved (at least between *C. auris*, *C. haemulonii* species complex, and *C. lusitaniae*) mating type locus (MTL) is present in the *C. auris* genome, consisting of a1 and a2 genes in MTLa isolates and a single α1 gene in the MTLα isolates (Muñoz et al., 2018). Interestingly, Clade I and Clade IV of *C. auris* isolates have so far all been MTLa, while Clade II and Clade III have all been MTLα. The mating type of Clade V has not yet been reported. The presence of a conserved mating type suggests that *C. auris* can mate, despite a lack of clear recombinants between or within clades.

Genomic variation between and within clades of *C. auris* may have an impact on gene function. The four clades of *C. auris* share an average pairwise nucleotide identity of 98.7% (Muñoz et al., 2018). In terms of nucleotide diversity (π), the four clades of *C. auris* had a value of 0.0039 which is less than *C. neoformans var. grubii* and more than the exclusively clonal *Trichophyton rubrum* (Muñoz et al., 2018). In terms of microevolution, Chow et al. (2018) found between 0 and 12 SNPs among clinical and screening cases in the United States within a patient, and also similar numbers within the facility during an outbreak. Comparisons between *C. auris* and its closest relatives showed changes to genes linked to drug resistance and virulence, including expanded families of transporters and lipases (Muñoz et al., 2018). However, much work remains to study positive selection between clades, and various features of population genetics within clades – including the identification of crossovers, effective population sizes, fixation indices, or evidence for bottlenecking.

Given the rapid clinical emergence of *C. auris*, including the newly identified Clade V in 2018 (Chow et al., 2019), the allelic variation within the species remains unclear. Further sampling of *C. auris* is clearly necessary, both in terms of screening historic samples that may have been mischaracterized, as well as new sampling efforts in both clinical and environmental settings. The discovery of additional clades, and the identification of clades with multiple mating types would assist in understanding the evolution of *C. auris*. A greater understanding of *C. auris*’ phylogenetics and population genetics would also be achieved by additional bioinformatic analysis – including a comprehensive characterization of gene gain/loss events and selection of alleles between or within those species or clades.

## Genomic Epidemiology of *C. auris*

Since 2009, five genetically diverse clades have been discovered from India and Pakistan (South Asian; Clade I), Japan (East Asian; Clade II), South Africa (African; Clade III), Venezuela (South American; Clade IV) (Lockhart et al., 2017b) and most recently in 2019, Iran (Clade V) (Chow et al., 2019). Over the past decade, isolates of *C. auris* have also been detected across all major continents, including elsewhere in Asia, Europe, the Middle East, Africa, Australia, and North and South America (Table 1). This nearly simultaneous and recent independent emergence is both unprecedented and raises questions regarding its origins and transmission events.

**TABLE 1:** *C. auris* has caused outbreaks across all major continents over the last decade.

Region	Country	Details	Clade(s)	Date	Citation
Asia	China	15 patients, 35 isolates (26 from urine, 4 catheter, 3 sputum, 1 blood, 1 fluid)	III	2011–2017	Tian et al., 2018
	India	3 hospitals, 19 isolates	I	2012–2015	Lockhart et al., 2017b
	Japan	1 patient (external ear canal)	II	2009	Satoh et al., 2009
	Malaysia	1 hospital, 1 patient	Unknown	2017	Mohd Tap et al., 2018
	Pakistan	2 hospitals, 18 isolates	I	2010–2015	Lockhart et al., 2017b
	Singapore	1 hospital, 3 patients (all with recent hospitalizations in India or Bangladesh)	Unknown	2012–2017	Tan and Tan, 2018
	South Korea	61 patients (4 blood, 57 ear) from 13 hospitals	II	1996–2018	Kwon et al., 2019
Europe	Austria	1 case	Unknown	2018	Kohlenberg et al., 2018
	Belgium	1 case	Unknown	2013–2017	Kohlenberg et al., 2018
	France	2 cases	Unknown	2013–2017	Kohlenberg et al., 2018
	Germany	7 cases (6 from patients previously hospitalized abroad)	I, III	2015–2017	Hamprecht et al., 2019
	Norway	1 case	Unknown	2013–2017	Kohlenberg et al., 2018
	Russia	1 hospital (49 cases in an ICU)	I	2016–2017	Barantsevich et al., 2019
	Spain	79 isolates, 738 environmental samples	Unknown	2017–2019	Ruiz-Gaitán et al., 2019
	Switzerland	1 patient who was on holiday in Spain, transferred to Switzerland hospital	Unknown	2017	Riat et al., 2018
	The Netherlands	2 cases (both from patients previously hospitalized in India)	I	2019	Vogelzang et al., 2019
	United Kingdom	72 patients (colonization, candidaemia, vascular lines)	I	2015–2016	Rhodes et al., 2018b
Middle East	Iran	1 patient	V	2018	Chow et al., 2019
	Israel	2 hospitals, 5 patients	Unknown	2014–2015	Ben-Ami et al., 2017
	Kuwait	56 patients, 158 isolates	Unknown	2014–2017	Khan et al., 2018
	Oman	2 patients	Unknown	2016–2017	Mohsin et al., 2017
	Saudi Arabia	1 hospital, 2 patients	I	2017–2018	Abdalhamid et al., 2018
	United Arab Emirates	1 hospital, 1 patient	Unknown	2017	Alatoom et al., 2018
Africa	Kenya	1 patient	III	2012	Heath et al., 2019
	South Africa	1,576 cases	III	2012–2016	Govender et al., 2018
Australia	Australia	1 patient (a 65-year old with recent hospitalization in Kenya)	III	2015	Heath et al., 2019
Americas	Canada	1 patient (a 64-year old with a recent hospitalization in India)	Unknown	2017	Schwartz and Hammond, 2017
	Columbia	4 hospitals, 7 people colonized, 37 environmental samples	IV	2015–2016	Escandón et al., 2019
	Panama	1 hospital, 9 patients, 14 isolates	Unknown	2016	Araúz et al., 2018
	United States	10 US states (133 isolates; 73 clinical cases and 60 screening cases)	I, II, III, IV	2013–2017	Chow et al., 2018
	Venezuela	1 hospital, 5 isolates	IV	2012–2013	Lockhart et al., 2017b

Genome sequencing of isolates from clinical environments is revealing detailed insights into how *C. auris* is spreading between continents, within countries, and even within wards of hospitals and over time in patients. For example, between 2013 and 2017, 133 isolates (73 clinical cases and 60 screening cases) from ten US states were collected and evaluated by whole genome sequencing (Chow et al., 2018) and compared to standardized case report forms and contact investigations, including travel history and epidemiological links. Chow et al. found that isolates were related to South Asian, South American, African and East Asian isolates, indicating multiple clades of *C. auris* were introduced into the United States, some of which perhaps several times. Surprisingly however, only 7% of the clinical cases had clear evidence of being acquired through health-care exposures abroad, suggesting travel data were lacking, the index patient with exposure were not identified, or that the infection many may have been acquired from other sources, perhaps including environmental sources. Chow et al. found a similarly small number of SNPs (between 0 and 12) among clinical and screening cases within a patient compared with those within a facility during an outbreak. The low numbers of genomic variants indicate that there is local and ongoing transmission of *C. auris* in the United States, with most (82%) of the clinical isolates reported from hospitals located in New York and New Jersey (Chow et al., 2018; Tracking Candida auris | CDC, 2020).

Outbreaks in Europe and Australia have also been attributed to recent spread from other continents. For example, of the 7 cases of *C. auris* identified in Germany during 2015–2017, 6 were from patients previously hospitalized abroad (Hamprecht et al., 2019). Whole-genome sequencing and epidemiologic analyses revealed that all patients in Germany were infected with different strains: five related to isolates from South Asia (Clade I), and one related to isolates from South Africa (Clade III) (Hamprecht et al., 2019). More recently (2019), the first two cases of *C. auris* were reported in The Netherlands, with both cases arising in patients that were treated in a healthcare facility in India prior to admission. Indeed, genetic analysis showed that these isolates also belonged to the South Asian *C. auris* Clade I (Vogelzang et al., 2019). In Australia in 2015, a 65-year-old man with a history of intensive care treatment in Kenya in 2012, was diagnosed with the South African Clade III of *C. auris* causing sternal osteomyelitis (Heath et al., 2019).

The largest outbreak in the United Kingdom to date of *C. auris* occurred between April 2015 and November 2016 in the Royal Brompton hospital in London, which involved 72 separate patients (Schelenz et al., 2016). Using Oxford Nanopore and Illumina sequencing technologies to gather genomic data on those clinical isolates, Rhodes et al. placed the United Kingdom outbreak in the India/Pakistan clade (Clade I), demonstrating an Asian origin for the outbreak. Rhodes et al. (2018a) were also able to estimate the timing for the most recent common ancestor (MRCA) of those outbreak strains to late March 2015, which was just weeks prior to the first patient identified with a *C. auris* infection. Additionally, by using root-to-tip regression, Rhodes et al. (2018a) estimated the evolutionary rate of the *C. auris* nuclear genome to 5.7 × 10^–5^ substitutions per site per year, a slower rate to the nuclear DNA of other related fungal species in the Saccharomycetales such as *Saccharomyces cerevisiae* (5.7 × 10^–3^), as well as more distantly related species such as the *Schizosaccharomyces pombe* traditional beer strains (3.0 × 10^–3^).

Multiple outbreaks of *C. auris* in Colombia between 2015 and 2016 led to a concerted effort to understand its epidemiology within Colombian health-care clinics (Escandón et al., 2019). By sampling infected patients, patient contacts, healthcare workers, and the environment across 4 hospitals, they identified likely sources of transmission events, including health-care workers, and in the hospital environment. For all studied groups, the swabbed body sites included: axillae, groins, nostrils and ears. Samples were also taken from the mouth and rectum of patients. Among patients, the axilla, groin, and rectum had the highest positivity rate (2/7; 28% each). *C. auris* was isolated from the hands of two healthcare workers and the groin of another healthcare worker (Escandón et al., 2019). None of the 4 patient contacts swabbed were positive for *C. auris*, although the low sample size does not preclude this as a mechanism of spread. *C. auris* was isolated from 37 of 322 (11%) environmental samples, including a diverse range of hospital equipment including on a stethoscope, hospital floor, body cables, bed railing, mattresses, stretchers, bedpans, a closet, towel, cell phone, mops, TV control, meal table, sink, toilet, wheelchair, and a nurse’s shoe. The results demonstrate that contamination can occur, and also that *C. auris* can survive on a variety of substances, at least in the short term (Escandón et al., 2019). Unlike the aforementioned European outbreaks, whole-genome sequencing and phylogenomics showed that the genetic background of isolates throughout the four hospitals were all similar (suspected Clade IV), despite the four hospitals spanning 700 km across Colombia. Isolates from the two northern Colombian hospitals grouped into a sub-clade, while those from the two southern hospitals grouped into a separate sub-clade, suggesting some geographically defined population structure in the country, and possibly endemism.

Since it was first discovered in Tokyo Metropolitan Geriatric Hospital (Tokyo, Japan) in 2009 (Satoh et al., 2009), *C. auris* has caused hospital acquired infections and outbreaks across all main continents including Asia, Europe, Africa, Australia, North America and South America. Given the recency of these outbreaks, further outbreaks across the world in the upcoming decade appear likely, unless concerted mitigation efforts are made (Escandón et al., 2019; Ong et al., 2019). It is also likely that cases are being misidentified (Chatterjee et al., 2015; Govender et al., 2018) or undetected, especially across Sub-Saharan Africa, South America and Eastern Europe where there are currently few reports (Ong et al., 2019). Exemplifying this is how common *C. auris* has become in some hospitals from regions with few additional reports of cases (van Schalkwyk et al., 2019). For example, in the Aga Khan University Hospital in Nairobi, Kenya between 2010 and 2016, *C. auris* was the most common cause of candidemia (38% of 201 patients) (Adam et al., 2019), suggesting that other hospitals in Kenya and in neighboring countries are likely to also harbor *C. auris*. Necessary steps to better understanding the epidemiology of *C. auris* will therefore include greater sampling and reporting from regions that underreported *C. auris* cases, a transition away from unreliable diagnostic methods, and further genomic analysis describing the population genetics of the various lineages, all of which may reveal its geospatial origin and subsequent spread.

## The Emergence of *C. auris*

To date, the earliest reports of *C. auris* have been from a Clade II isolate in 1996, retrospectively from a case of misidentified nosocomial fungemia in South Korea (Lee et al., 2011). The species was first described over 10 years later from a case in Japan (Satoh et al., 2009), and simultaneously arising in South Africa with a Clade III isolate also from 2009 (Govender et al., 2018). *C. auris* has not been detected earlier than 2009 by The SENTRY Antifungal Surveillance Program, which has been collecting consecutive invasive *Candida* isolates from 135 participating medical centers in North America, Europe, Latin America and the Asia-Pacific regions since 1997 (Pfaller et al., 2019). However, molecular clock estimates from 304 worldwide genomes date the most recent common ancestor of each of the four clades within the last 339 years, and outbreak isolates from Clades I, III, and IV to 34–35 years ago (∼10 years prior to first being detected) (Chow et al., 2020). While the details of its origin remain unclear, *C. auris* is not the only fungal pathogen to have recently clinically emerged. Indeed, a variety of phylogenetically diverse emerging fungal pathogens are rapidly increasing in their incidence, geographic or host range and virulence (Morse, 1995; Farrer and Fisher, 2017). Recently highlighted examples include the emergence and increasing incidence of a separate ascomycete responsible for human infections: *Emergomyces africanus* (Dukik et al., 2017), the newly-described chytrid fungus *Batrachochytrium salamandrivorans* causing rapid declines of fire salamanders across an expanding region of northern Europe (Farrer, 2019), and the basidiomycete fungus *Cryptococcus gattii* expanding its range into non-endemic environments with a consequential increase of fatal disease in humans (Fraser et al., 2005; Byrnes et al., 2010).

One suggestion for the absence of *C. auris* isolates prior to 1996 is that it has only recently become part of the human microbiome, and has either recently acquired sufficient virulence to cause invasive infections or been newly introduced into human populations in which invasive *Candida* infections can be identified (Jackson et al., 2019). For example, recent genetic recombination, hybridization, or other biological changes could potentially increase the organism’s transmissibility or virulence (Jackson et al., 2019). However, for this hypothesis to hold, such genetic changes would need to have occurred across all clades of *C. auris* recently.

One line of inquiry to understand the recency of the emergence has been to re-screen isolates taken prior to 2009 that may have been misdiagnosed as other *Candida* species. Indeed, *C. auris* has been misdiagnosed as its closest relatives *C. haemulonii* or *C. pseudohaemulonii* (Chatterjee et al., 2015), species which have even been identified alongside *C. auris* in the same month in the same hospital (Ben-Ami et al., 2017). Lee et al. (2011) identified the oldest known case of *C. auris* from South Korea, which dates to 1996, belongs to Clade II, and had been misidentified as *C. haemulonii* by Vitek 2. Another clinical isolate originally characterized as *C. haemulonii* from a hospital in South Africa, was later shown to be a Clade III isolate of *C. auris* (Govender et al., 2018). This isolate was taken from a patient in 2009 demonstrating independent emergences in 2009 of at least two of the *C. auris* lineages (Clade II and Clade III). A recent study comparing 204 genomes of *C. auris* using Bayesian molecular clock phylogenetics estimated the origin of each of the four clades within the last 339 years, and outbreak clusters (Clade I, III, and IV) originating 34–35 years ago (Chow et al., 2020), suggesting more historic samples were not identified.

The spread of *C. auris* within and between hospital settings has been clearly demonstrated by at least clades I, III and IV (e.g., Chow et al., 2018; Rhodes et al., 2018b; Escandón et al., 2019), so it is possible that abiotic factors could explain the emergence. For example, human activities such as deforestation, expansion of farmland, and coastal ecosystem disruption could have allowed an ecological jump (Jackson et al., 2019). Indeed, *C. auris*’ closest relatives include *C. ruelliae* from flowers of the *Ruellia* species (Saluja and Prasad, 2008), and *C. haemulonii* that can also cause clinical infections but also found in the GI tract and skin of marine animals (van Uden and Kolipinski, 1962; Khan et al., 2007). Furthermore, *C. auris* has been shown to persist on a wide variety of hospital equipment (Escandón et al., 2019). It therefore seems likely that *C. auris* may also have an environmental niche (plants or aquatic). The widespread use of fungicides, especially triazoles in agriculture could have been responsible for *C. auris* to become more prominent in the environment, as it shows high levels of antifungal resistance. At the same time, increased human travel, trade or other anthropogenic factors may have simply led to the globalization and emergence of *C. auris* (Jackson et al., 2019).

*C. auris* is able to grow (albeit slowly) at 42°C, while isolates from close relatives (*C. haemulonii*, *C. pseudohaemulonii* or *C. heveicola*) were unable to grow at this temperature (Satoh et al., 2009). *C. auris*’ thermotolerance enables it to cause invasive candidemia, including tolerating the fever response (Jackson et al., 2019), but may also enable it to sustain body temperatures of other animals including birds (Casadevall et al., 2019). For example, pigeons body temperature can be up to 44°C during flight (Aulie, 1971), compared with only a few degrees above 37°C during exercise for humans (Gleeson, 1998). Similarly, sea birds may serve as reservoirs for indirect transmission of drug-resistant *Candida* species, such as *C. glabrata*, to humans (Al-Yasiri et al., 2016; Casadevall et al., 2019). *C. auris* is also able to survive high salt concentrations (broth containing 10% NaCl), which might allow it survive environmentally including hypersaline desert lakes, salt-evaporating ponds, or tidal pools (Jackson et al., 2019). Thus, *C. auris* is thought likely to survive both in the environment, as well as colonizing human skin. On skin it is likely to compete with other commensals such as *Malassezia*, although studies on *C. auris* interactions with bacteria or other fungi are lacking.

The thermotolerance of *C. auris* has led to the hypothesis that its emergence may be linked to climate change and global temperature changes, and may even be the first example of a new pathogenic fungus emerging from human-induced global warming (Casadevall et al., 2019). Indeed, it has been proposed that as the gap between the environmental temperature and human body temperature narrows, new fungal diseases of mammals will increase (Garcia-Solache and Casadevall, 2010). Casadevall et al. (2019) suggest a number of factors that supports the hypothesis of a climate change emergence, including that *C. auris* can grow on skin but not anaerobically in the gut (suggesting a recent environmental source), the constitutive overexpression of heat shock protein HSP90, and the inability of many relatives to grow at high temperatures. *C. auris* clades I, II, III, and IV differ by an average of 1.3% average pairwise nucleotide identity (Muñoz et al., 2018). Although a time to most recent common ancestor of multiple clades has not yet been predicted, the ancestor to all clades almost certainly predates industrial era warming, which begun in 1800 AD (Abram et al., 2016). Given that all known clades of *C. auris* are able to colonize skin and cause infections, its ability to colonize these niches are more likely to be an evolutionary conserved trait, rather than five independent recent trait acquisitions. It nevertheless remains to be shown if climate change had a role to play in exposing each clade into an alternative niche they already had the ability to colonize (Casadevall et al., 2019).

Further environmental sampling, genome sequencing and metagenomics studies focusing on fungi are necessary to identify and confirm environmental sources of *C. auris*. Further genomic epidemiology studies, including on the genetic diversity within each clade will help to find older natural populations that could indicate the progenitor of *C. auris*. Clade II (East Asian clade) exhibits higher genetic diversity than the other clades (Jackson et al., 2019), although it also exhibits large karyotypic variation and loss of sub-telomeric regions, suggesting it is not ancestral to Clade I, III and IV. The discovery of further ecological niches and the geographical origin will be important to prevent similar emergences in future as well as identify and mitigate emergence of new clades of *C. auris* that have the potential to cause outbreaks.

## Antifungal Treatment of *C. auris*

Treating invasive fungal infections is a considerable challenge due to the limited number of available antifungal agents. The five major antifungal drug classes that are used in hospital settings are azoles, allylamines, polyenes, echinocandins and nucleoside analogs (Bidaud et al., 2018). To date, there are only a few alternative antifungal agents (de Oliveira Santos et al., 2018). While the efficacy of some antifungal classes is highly dependent on the site of infection due to their tissue penetration properties (Felton et al., 2014), other classes of antifungals (i.e., polyenes) often lead to serious side effects such as renal injury or cardiomyopathy (Bandeira et al., 2016), primarily due to conserved or structural similarities in drug targets such as cholesterol and ergosterol (Kamiñski, 2014). Given the lack of treatment options, the emergence of fungal resistance to even one of the major drug classes is very alarming, as it makes the infection considerably more challenging to treat.

It is of considerable concern to health authorities that the emergent *C. auris* includes isolates that are resistant to all antifungals (Warris, 2018). *C. auris* isolates are resistant to fluconazole, the major drug in treating candidemia, with MIC values > 64 μg/mL (Chowdhary et al., 2018; Warris, 2018). Other azoles such as voriconazole show variable antifungal activity (Table 2). While more than 95% of isolates from India were resistant to a topical allylamine, terbinafine (Chowdhary et al., 2018), currently UK strains remain susceptible to terbinafine and polyene nystatin (Sarma and Upadhyay, 2017). Almost one-third of isolates observed so far were resistant to amphotericin B (a polyene used as a last resort drug) (Warris, 2018). Although the nucleoside analog 5-flucytosine successfully treated more than 95% of *C. auris* infection cases *in vitro* (Lockhart et al., 2017a; Osei Sekyere, 2018), it is not used extensively as therapy because resistance arises rapidly, potentially during treatment (Rhodes et al., 2018a), and it elicits toxic effects on bone marrow that may lead to death in immunosuppressed patients (Vermes et al., 2000). Between 2 and 7% of *C. auris* isolates have developed resistance to echinocandins (Lockhart et al., 2017b; Chowdhary et al., 2018), one the newest classes of antifungal drug to be developed (Sucher et al., 2009). Reported adverse effects of echinocandin therapy are generally mild and include nausea and dose-related elevation of liver aminotransferases levels (Aguilar-Zapata et al., 2015) and therefore, this group of drugs is still the most effective for treating the majority of *C. auris* infections. Resistance to all three common antifungal classes used as therapy (azoles, polyenes and echinocandins) was observed in 4% of *C. auris* outbreak samples in North America from 2012–2015 (Lockhart et al., 2017b).

**TABLE 2:** *Candida auris* resistance patterns.

				Samples	Resistance threshold	
Drug category	Drug	Method	Resistant	no	used (μg/mL)	References
Azoles	Fluconazole	CLSI	93%	54	32	Lockhart et al., 2017b
		CLSI	86%	123	32	Arendrup et al., 2017
		EUCAST	96%	123	32	Arendrup et al., 2017
		CLSI	90%	320	32	Chowdhary et al., 2018
	Voriconazole	CLSI	54%	54	2	Lockhart et al., 2017b
		CLSI	33%	123	2	Arendrup et al., 2017
		EUCAST	15%	123	2	Arendrup et al., 2017
		CLSI	39%	90	2	Kathuria et al., 2015
		Vitek 2	29%	90	2	Kathuria et al., 2015
		Etest	28%	90	2	Kathuria et al., 2015
		CLSI	15%	320	2	Chowdhary et al., 2018
	Isavuconazole	CLSI	4%	123	2	Arendrup et al., 2017
		EUCAST	4%	123	2	Arendrup et al., 2017
	Itraconazole	CLSI	0%	54	2	Lockhart et al., 2017b
		CLSI	1%	123	2	Arendrup et al., 2017
		EUCAST	0%	123	2	Arendrup et al., 2017
		CLSI	6%	320	1	Chowdhary et al., 2018
	Posaconazole	CLSI	0%	54	2	Lockhart et al., 2017b
		CLSI	2%	123	2	Arendrup et al., 2017
		EUCAST	0%	123	2	Arendrup et al., 2017
Polyenes	Amphotericin B	CLSI	35%	54	2	Lockhart et al., 2017b
		CLSI	16%	90	2	Kathuria et al., 2015
		Vitek 2	100%	90	2	Kathuria et al., 2015
		Etest	1%	90	2	Kathuria et al., 2015
		CLSI	8%	320	2	Chowdhary et al., 2018
		CLSI	10%	123	2	Arendrup et al., 2017
		EUCAST	0%	123	2	Arendrup et al., 2017
	Nystatin	CLSI	100%	320	2	Chowdhary et al., 2018
Echinocandins	Anidulafungin	CLSI	2%	320	8	Chowdhary et al., 2018
		CLSI	6%	123	8	Arendrup et al., 2017
		EUCAST	0%	123	8	Arendrup et al., 2017
		CLSI	7%	54	8	Lockhart et al., 2017b
	Caspofungin	CLSI	2%	320	8	Chowdhary et al., 2018
		CLSI	3%	90	8	Kathuria et al., 2015
		Vitek 2	0%	90	8	Kathuria et al., 2015
		Etest	0%	90	8	Kathuria et al., 2015
		CLSI	7%	54	8	Lockhart et al., 2017b
	Micafungin	CLSI	2%	320	8	Chowdhary et al., 2018
		CLSI	7%	54	8	Lockhart et al., 2017b
		CLSI	6%	123	8	Arendrup et al., 2017
		EUCAST	6%	123	8	Arendrup et al., 2017
Nucleoside analogs	5-Flucytosine	CLSI	6%	54	128	Lockhart et al., 2017b
Allylamines	Terbinafine	CLSI	100%	320	2	Chowdhary et al., 2018

**Prior to publishing C. auris tentative resistance thresholds, various research groups used different concentrations to determine the resistance of their isolates. Their choices were based on a comparison of the treatment outcomes associated with drug exposure observed in C. auris and in other Candida spp. infections.**

Although *C. auris* is typically multidrug-resistant, levels of susceptibility to various drugs differ greatly between isolates and clades (Kean and Ramage, 2019). To find the most suitable drug candidate, susceptibility is typically measured over the period of 24 h, when the fungus grows in the presence of a drug concentration gradient. If the observed minimal inhibitory concentration (MIC) is greater than the resistance MIC breakpoint, the examined isolate is considered resistant to the drug. Tentative susceptibility MIC breakpoints for *C. auris* established by CDC are shown in Table 3. Currently, these *C. auris* susceptibility breakpoints should be treated as a general guide as the connection between them and clinical outcome is not known yet.

**TABLE 3:** Tentative MIC breakpoints of *C. auris* defined by CDC (Antifungal Susceptibility Testing and Interpretation, 2019).

Drug class	Drug	Tentative MIC breakpoint (μg/mL)
Azoles	Fluconazole	≥32
Azoles	Other azoles	N/A
Polyenes	Amphotericin B	≥2
Echinocandins	Anidulafungin	≥4
Echinocandins	Caspofungin	≥2
Echinocandins	Micafungin	≥4

Importantly, some *Candida* such as *C. albicans* exhibit drug tolerance defined as the ability of a fraction of a population to grow above the population resistance level (Rosenberg et al., 2018). The presence of highly tolerant sub-populations may lead to persistent candidemia that is associated with a mortality rate higher than 50% (Hammoud et al., 2013) despite appropriate treatment (Alatoom et al., 2018). *In vivo* data suggest that reducing drug susceptibility without affecting the resistance of the population may be possible using adjuvant drugs (Rosenberg et al., 2018). Adjuvants such as the antidepressant fluoxetine that impairs biofilm development (Oliveira et al., 2018) or Hsp90 inhibitor called radicicol that blocks stress responses (Rosenberg et al., 2018) have been shown to clear tolerance without altering the susceptibility level when given in combination with fluconazole in *C. albicans* (Rosenberg et al., 2018). Future clinical control trials are required to confirm the effectiveness of adjuvants before being considered for inclusion as a possible treatment protocol.

## Mechanisms of Drug Resistance in *C. auris*

**FIGURE 1:**
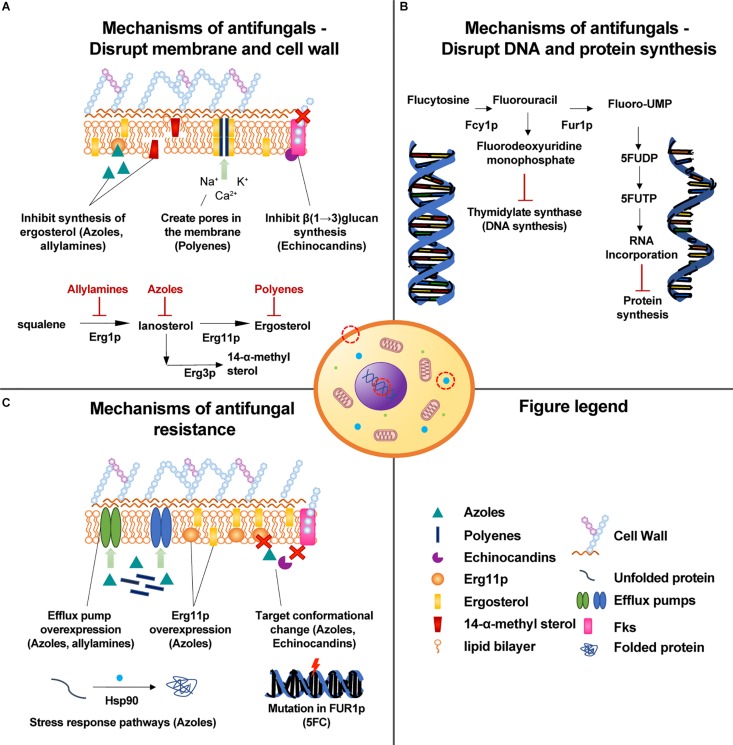
Mechanisms of drug action and resistance observed in *C. auris*. **(A)** The main mechanisms of antifungals that disrupt the cell membrane or cell wall. **(B)** In the nucleus, 5-flucytosine inhibits the synthesis of fungal DNA and RNA. **(C)** Mechanisms of antifungal resistance to drugs that damage the cell membrane or cell wall.

*C. auris* has evolved a range of molecular drug-resistance mechanisms which are shown in Figure 1. They include (1) Drug target mutation (Bidaud et al., 2018), (2) drug target overexpression (Bhattacharya et al., 2019), (3) changes in drug uptake and efflux (Muñoz et al., 2018), (4) activation of stress response pathways (Kim et al., 2019), and (5) biofilm formation (Kean et al., 2018). By aggregating into a colony and forming a biofilm, *Candida* spp. increase their resistance to all currently available antifungals by up to 1000-fold (Taff et al., 2013). Some of the key regulators of fungal biofilm dispersion, antibiotic tolerance, and cell wall remodeling are molecular chaperones belonging to Hsp90 family (Robbins et al., 2011; Leach et al., 2016). In *C. auris*, these proteins were found to promote cell-wall integrity signaling and stress responses related to azoles administration thereby contributing to the evolution of drug resistance (Kim et al., 2019).

Azoles are an important group of antifungal agents that were first developed in the late 1960s, and function by inhibiting the synthesis of ergosterol, a key component of the fungal membrane, thereby preventing growth and proliferation (Ghannoum and Rice, 1999). Since the action of azoles is largely dependent on the affinity of the drug to the active site of Erg11p, any mutations that affect the active site of the enzyme may lead to the development of drug resistance (Berkow and Lockhart, 2017). Such resistance mechanisms have been observed in *C. albicans*, in which more than 140 ERG11 mutations have been identified to date (Debnath and Addya, 2014). These mutations accumulated in three regions of the ERG11 gene (Marichal et al., 1999). Bioinformatic analysis of these substitutions combined with 3D modeling showed that most of them are located near the predicted catalytic center or on elements of the fungus-specific external loop (Whaley et al., 2017). Many of the described mutations can also be found in *C. auris*, but not all have the same effects (Muñoz et al., 2018). In both species, amino acid substitutions at F126L, Y132F, K143R result in azole resistance (Morio et al., 2010; Chowdhary et al., 2018; Healey et al., 2018), with Y132F the most widespread mutation associated with azole resistance (Chow et al., 2020). Another mechanism of azole resistance is the overexpression of Erg11p, which can be caused either by increasing the number of ERG11 transcription factors such as Upc2p or by duplicating ERG11 (Bhattacharya et al., 2019). In both cases, the high concentration of azole target dilutes the effects of drug activity (Muñoz et al., 2018).

The activity of azoles can be also reduced by decreasing the concentration of antifungals in the cell by means of efflux pumps. Recently, 20 ABC transporters that are putative efflux pumps have been identified in the *C. auris* genome, strain-type CBS 10913T (Wasi et al., 2019). Two drug transporters (CDR1 and MDR1) that have primarily been studied in *C. albicans* have orthologs in *C. auris*, and these orthologs are overexpressed in azole-resistant *C. auris* (Rybak et al., 2019). CDR1 is an ABC transporter that confers azole-derived compounds resistance, while MDR1 is a Major Facilitator Superfamily (MFS) pump and a member of the multidrug resistance family which is responsible for fluconazole resistance (de Oliveira Santos et al., 2018). Rybak et al. demonstrated that deletion of CDR1 restored susceptibility of highly resistant *C. auris* isolates to fluconazole. Furthermore, domains associated with transporters such as OPT and glutathione were found to be significantly enriched in *C. auris*, *C. haemulonii*, *C. duobushaemulonii*, and *C. pseudohaemulonii* when compared to related species (Muñoz et al., 2018). Oligopeptide transporters (OPT) are small peptide transporters expression of which is upregulated in *C. albicans* upon administration of azoles (Muñoz et al., 2018). Similarly, glutathione transporters may contribute to azole resistance by exporting oxidized glutathione derivatives out of the cell and thereby protecting the cell against oxidative damage elicited by the drugs (Maras et al., 2014).

Polyenes are a commonly used group of drugs used to treat *C. auris* infections. The most known polyene, amphotericin B, binds membrane-bound ergosterol, causing pores to form in the fungal membrane through which small molecules leak to the outside of the cell (de Oliveira Santos et al., 2018). The release of intracellular constituents such as sugars, potassium or calcium strongly contributes to cell death (de Oliveira Santos et al., 2018). We are not aware of any mechanism of polyenes resistance reported for *C. auris* so far, which would presumably be analogous to ERG3 dependent alternations in sterol synthesis observed in *C. albicans* or alternations in cell membrane sterol composition (Taff et al., 2013). However, in 2018, Muñoz et al. (2018) showed that intrinsic transcription of multidrug transporters in *C. auris* was higher when compared to susceptible and resistant isolates and that upon administering Amphotericin-B, eight genes including OPT1-like transporter (high-affinity glutathione, tetra- and pentapeptides transporter), CSA1 (conserved cell wall protein 1 involved in biofilm formation), MET15 (sulfhydrylase) and ARG1 (argininosuccinate synthase) were upregulated. In 2019, another study identified five novel mutations that associate strongly with amphotericin B resistance in Colombian *C. auris* (*p* ≤ 0.001). Four of the identified mutations fall within protein-coding regions (Escandón et al., 2019). One of the mutations occurs within FLO8, a transcription factor that is required for virulence and biofilm formation in *C. albicans* (Escandón et al., 2019). Another one is located within a predicted transmembrane protein that could contribute to drug resistance (Escandón et al., 2019). Drug transporters overexpression upon administering amphotericin B was also observed by Wasi et al. In their study, they found 8.7-fold increase in expression of a homolog of CDR6 ABC transporter (Wasi et al., 2019). In the search for more robust drugs and drug targets, we urgently need to better understand the structure and function of drug pumps in pathogenic fungi, as well as how allelic diversity and mutations can affect drug efficacy.

Echinocandins act through the non-competitive inhibition of FKS1 gene product called β(1→3) glucan synthase, which results in stopping production of glucan. Because of depleted glucan, the fungal cell becomes weak and prone to osmotic stress (de Oliveira Santos et al., 2018). *C. auris*’ resistance to echinocandins has been linked to mutations S639F, S639P and S639Y that occur in the highly conserved hot-spot 1 of FKS1 and decrease the sensitivity of the enzyme to the drug (Bidaud et al., 2018; Hou et al., 2019). S639P in FKS1 was recently shown to be the most widespread mutation associated with echinocandin resistance (Chow et al., 2020). A recent multi-omics analysis revealed that although the cell wall remodeling enzymes concentration was lower in *C. auris* when compared to *C. albicans*, in a drug-resistant *C. auris* isolate (MIC fluconazole: > 256 μg/mL, MIC caspofungin: 2 μg/mL) cell wall integrity proteins were upregulated, suggesting that the cell wall metabolism can be adjusted in response to drug treatment (Zamith-Miranda et al., 2019).

Little is known about *C. auris* resistance mechanisms to other drug classes such as nucleoside analogs or allylamines (Jeffery-Smith et al., 2018). The nucleoside analogs class contains only 5-flucytosine (5FC) – a drug inhibiting RNA and DNA synthesis in pathogenic fungi (Rapp, 2004). One amino acid substitution (F211I) that is associated with 5FC resistance has been identified in the conserved FUR1 sequence encoding uracil phosphoribosyltransferase (Rhodes et al., 2018b). However, further work is required to understand how such polymorphisms may affect the functioning of the enzyme, which is responsible for the conversion of 5-fluorouracil into 5-fluoro-UMP acid monophosphate. For allylamines, a homolog of ABC transporter CDR6 was found to be significantly upregulated in drug-resistant *C. auris* following terbinafine treatment (Wasi et al., 2019).

## Detection and Novel Treatments of *C. auris*

Successful treatment of *C. auris* infection depends heavily on its accurate identification. In the early stages of infection, symptoms are non-specific and blood cultures typically remain negative (Pfaller and Castanheira, 2016). The resulting delay in diagnosis directly translates to decreased chances of survival; in the study of *C. auris* outbreak in a European hospital between 2016 and 2017, 41% of patients died within 1 month from infection (Ruiz-Gaitán et al., 2018).

*C. auris* does not possess phenotypic features that could easily distinguish it from other *Candida* spp. (Osei Sekyere, 2018). Depending on the strain it appears purple, red, pink or white on CHROMagar (Kordalewska and Perlin, 2019b) but unlike most types of yeast, it is able to withstand high temperatures (42°C) (Osei Sekyere, 2018). Many misidentifications related to automated tools such as VITEK 2, BD Phoenix, RapID Yeast Plus, API 20C have been reported to date. Ambaraghassi et al. (2019) demonstrated that VITEK 2 was unable to consistently identify *C. auris* and that the identification depended on the clade. While the tool identified correctly the South American clade (*n* = 8), its accuracy was variable for isolates from the African (7%, *n* = 10) and East Asian clades (0%, *n* = 4) (Ambaraghassi et al., 2019). Genomics analysis showed that the low identification rate was likely due to the deletion of the L-rhamnose 7-gene cluster in all clades except for the South African clade (Ambaraghassi et al., 2019). As one of 20 tests performed by the VITEK 2 platform is based on the ability of the tested organism to assimilate L-rhamnose (Graf et al., 2000, 2), the deletion of L-rhamnose gene cluster can have an impact on identification results. The most reliable way to identify *C. auris* without genomic sequencing is MALDI-TOF MS (Vatanshenassan et al., 2019). Although not all reference databases contain its protein profile, a free supplemental update is available from MicrobeNet (Lockhart et al., 2017a). A variety of groups have also had success with sub-genomic methods such as sequencing D1-D2 region of 28s ribosomal DNA (rDNA) or internal transcribed spacer region of rDNA (Jeffery-Smith et al., 2018). Unfortunately, access to such tools is limited in many community hospitals.

The necessity for expeditious identification of *C. auris* in order to effectively treat patients and prevent outbreaks combined with slow current standard laboratory techniques creates a pressing need for new diagnostic tools that could rapidly recognize *C. auris* and its drug resistance patterns. Most of biochemical automated systems and MALDI-TOF MS require cultures, which can take up to 2 weeks to obtain (Kordalewska and Perlin, 2019a). To identify *C. auris* directly from skin swabs, molecular-based assays such as loop-mediated isothermal amplification (LAMP), T2 magnetic resonance and PCR/qPCR have been applied (Kordalewska and Perlin, 2019b). Since DNA can be isolated directly from the clinical sample, these tests can deliver diagnostic results within several hours (Kordalewska and Perlin, 2019a), which dramatically increases the probability of survival, given that the time window appropriate for initiating appropriate antifungal therapy in *Candida* bloodstream infection was estimated to 12 h (Morrell et al., 2005).

The choice of optimal *C. auris* treatment method depends on both the patient (age, previous use of antifungal medications, infection site, immune status) and the attacking pathogen (strain, possible biofilm formation, drug resistance patterns) (Dodgson et al., 2004; Felton et al., 2014; Bidaud et al., 2018; Moser et al., 2019). The mainstay of therapy is antibiotics. In adults, it is encouraged to begin the therapy with echinocandins even before receiving susceptibility test results (Bidaud et al., 2018). Amphotericin B should be considered for neonates, infants below 2 months of age and as a single or in combination with micafungin when monotherapy with echinocandins fails (Alfouzan et al., 2019; Park et al., 2019). Among azoles, the highest antifungal activity has been observed for itraconazole, isavuconazole and posaconazole (Bidaud et al., 2018), which are sometimes used as a secondary therapy. When voriconazole and micafungin are taken together they show greater effects when compared to the sum of the effects of these drugs taken separately (Cortegiani et al., 2018). While systemic antibiotic treatment is not advised for people colonized with *C. auris*, control measures should be followed in all cases (Bidaud et al., 2018). Invasive devices such as catheters and ventricular shunts should be removed as soon as they are no longer needed as they may be infected with a biofilm (Bidaud et al., 2018).

*C. auris* develops resistance rapidly while the patient is being treated and therefore it is crucial to use the available antifungals for the right amount of time and in the right dose (Jeffery-Smith et al., 2018). Close monitoring of drug susceptibility is necessary in both infection and colonization cases in order to identify changes in antifungal resistance (Bidaud et al., 2018). Furthermore, special care is required when administering fungistatic drugs as they may provide the opportunity for resistance to emerge (Berkow and Lockhart, 2017). A small number of new anti-fungal agents are showing successes in clinical trials that may become available for wider use in hospital settings in the upcoming years (Table 4).

**TABLE 4:** New antifungals in trials.

Company	Drug	Trial (Phase)	Activity	References
Amplyx	Fosmanogepix (APX001)*	*C. auris* (Ib), Candidemia (II)	Gwt1 inhibitor (novel)	Hager et al., 2018
Synexis	Ibrexafungerp*	*C. auris* (III)	Glucan synthase inhibitor (novel, orally available)	Berkow and Lockhart, 2017; Larkin et al., 2019
NQP 1598	VT-1598	*C. auris* (I)	CYP51 (Erg11p) inhibitor	Wiederhold et al., 2019
Mycovia	VT-1161	Candidiasis (III)	CYP51 (Erg11p) inhibitor	Brand et al., 2018, p. 2
Cidara	Rezafungin	Candidemia (III)	Long half-life echinocandin	Lepak et al., 2018

*Fosmanogepix (Hodges et al., 2017) and Ibrexafungerp (Juneja et al., 2019) are available in an emergency in fungi research centers such as University Hospital Graz (Graz), University Hospital Innsbruck (Innsbruck), Radboud University (Nijmegen) and University Hospital of Manchester (Manchester).

## Mechanisms of Virulence in *C. auris*

**FIGURE 2:**
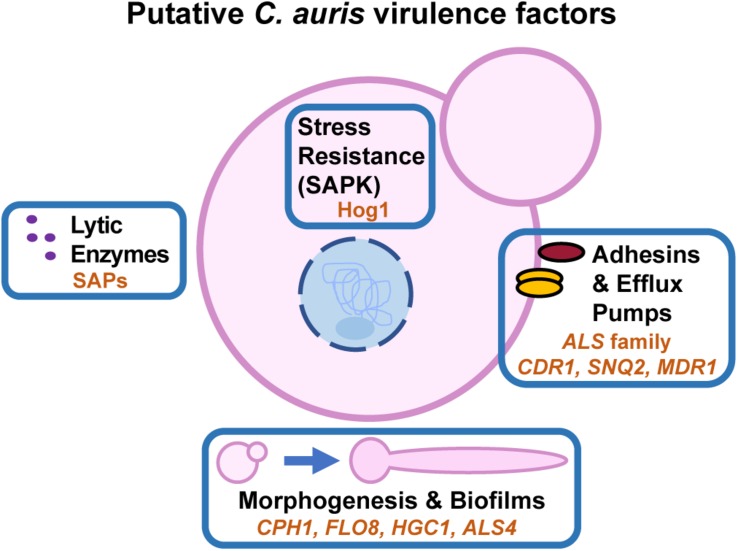
Putative *C. auris* virulence factors. Key orthologs of *C. albicans* virulence factors that are transcriptionally induced during *C. auris* growth at 37°C are indicated by orange text within each box. *C. auris* Hog1 is the only conserved ortholog with *C. albicans* that has been experimentally confirmed to play a role in virulence.

Stress resistance, thermotolerance, adherence to host cells, and immune evasion are important virulence traits across *Candida* species. Investigations of *C. auris* virulence traits often draw comparisons to the extensively-studied pathogen, *C. albicans*. The *C. auris* genome encodes several orthologs of known virulence factors in *C. albicans* including genes associated with biofilm formation, antifungal drug resistance, and phenotypic switching (Muñoz et al., 2018). Recent virulence, transcriptome, and other studies are highlighting how these conserved fungal virulence mechanisms may contribute to *C. auris’* emergence as a nosocomial pathogen (Figure 2).

### Virulence

Conflicting reports abound regarding *C. auris*’ virulence *in vivo* compared to other pathogenic *Candida* species. Depending on the isolate and infection model used, *C. auris* may be regarded as more virulent (Sherry et al., 2017), as virulent (Borman et al., 2016; Fakhim et al., 2018), or less virulent (Ben-Ami et al., 2017; Wang et al., 2018) than *C. albicans* in murine and invertebrate infection models. In studies utilizing the *Galleria mellonella* invertebrate infection model, *C. auris* isolates exhibit similar virulence phenotypes, or even slightly more virulent phenotypes, than *C. albicans* (Borman et al., 2016; Sherry et al., 2017).

Investigators have used immunocompetent and immunocompromised murine models of invasive candidiasis to evaluate *C. auris* virulence. In immunocompromised animals infected with Israeli *C. auris* clinical isolates, infection lead to rapid death within 5 days, but overall *C. auris* exhibited decreased virulence and lower kidney fungal burdens compared to *C. albicans* strain CBS 8837 (Ben-Ami et al., 2017). One study with immunocompetent animals established that two clade II *C. auris* isolates had similar virulence outcomes compared to one *C. albicans* clinical isolate (IFRC 1442) (Fakhim et al., 2018), while a study in China determined that the first *C. auris* isolate identified in China was significantly less virulent than the standard *C. albicans* laboratory strain SC5314 (Wang et al., 2018). To date, data from murine infection models are difficult to interpret due to inconsistencies in the *C. auris* and comparator *C. albicans* strains employed between studies. Consequently, it is not currently possible to speculate whether *C. auris* or individual clades of *C. auris* are generally more, less, or similarly virulent during mammalian infection compared to the prototypic pathogen *C. albicans*.

Phenotypic and genotypic variability between different *C. auris* isolates are plausible explanations for the discrepancies described in virulence studies. This variability is somewhat unsurprising given the broad geographical distribution and genetic diversity among clades of *C. auris* outbreaks, but also highlights our poor understanding of *C. auris* pathogenesis. Further characterization of virulence determinants, some of which will be discussed in this section, are important for understanding the virulence potential of this organism. The field would significantly benefit from standardizing virulence investigations by adopting one major *C. albicans* comparison strain and utilizing high-throughput *in vitro* methodologies to rapidly identify type strains for each *C. auris* clade that are suitable for comparative *in vivo* studies.

### Lytic Enzymes

Lytic enzymes, such as secreted aspartyl proteases (SAPs), lipases, phospholipases, and hemolysins, are important virulence factors in human pathogenic fungi (Chaffin, 2008). These enzymes facilitate host cell invasion and tissue destruction by *C. albicans* (Polke et al., 2015). *C. auris* encodes orthologs of many known *C. albicans* lytic enzymes, including secreted aspartyl proteases, secreted lipases, and phospholipases (Chatterjee et al., 2015). Several studies have demonstrated lytic activity for *C. auris* SAPs (Yue et al., 2018), phospholipases (Larkin et al., 2017), and hemolysin (Kumar et al., 2015), but little is known about how the expression profiles of these enzymes compare to their *C. albicans* orthologs *in vitro* or *in vivo.* Two SAP orthologs were transcriptionally induced in *C. auris* filamentous cells compared to yeast cells (Yue et al., 2018), but their relative roles in virulence remain unknown. Further molecular and virulence studies are necessary to determine the importance of lytic enzymes to *C. auris*’ infection strategy.

### Filamentous Growth

While filamentation is integral to *C. albicans* virulence (Zheng et al., 2004), its contribution to *C. auris* virulence is less certain given that *in vivo* evidence to date has identified only yeast cells in animal tissues (Ben-Ami et al., 2017). Interestingly, *in vivo* passage can confer a filamentation-competent (FC) phenotype, but FC and yeast cells have comparable virulence in mice suggesting that filamentation is dispensable for *C. auris* virulence (Yue et al., 2018).

*In vitro C. auris* filamentation occurs under a restricted and/or atypical set of growth conditions compared to *C. albicans* (Wang et al., 2018; Yue et al., 2018). Like *C. albicans*, Hsp90 depletion or pharmacological inhibition induces filamentous growth and the expression of adhesins and cell wall genes orthologous to *C. albicans* filament-associated genes (Kim et al., 2019). During FC-triggered filamentous growth, orthologs of *C. albicans CPH1* and *FLO8*, which encode transcriptional regulators of filamentous growth, were induced in *C. auris* filamentous cells compared to yeast (Yue et al., 2018). In addition, orthologs of *C. albicans* filament-associated genes were upregulated during *C. auris* filamentation, including the G1 cyclin-related gene *HGC1* and glycosylphosphatidylinositol (GPI) anchored gene *ALS4*. However, there were key differences in the filamentation transcriptomes for *C. auris* relative to *C. albicans. EFG1*, an important transcriptional regulator of filamentation in *C. albicans*, was down-regulated in *C. auris* filamentous cells. Other *C. albicans* filament-associated cell wall orthologs were preferentially expressed during *C. auris* yeast growth (*PGA34, PGA38*, and *PGA58*) (Yue et al., 2018). Thus, while *C. auris* shares some transcriptomic aspects of the *C. albicans* filamentous growth program, the role of *C. auris* filamentation and filament-associated genes in its pathogenicity requires further study.

### Adherence and Biofilm Formation

Adherence to host cells is essential to microbial colonization, persistence, and virulence (Mayer et al., 2013; Tsui et al., 2016). In addition, microbe-microbe adherence mediates the formation of microbial communities, or biofilms, which are an important virulence trait in *Candida* species that confer enhanced antimicrobial resistance. The *C. auris* genome encodes several orthologs of *C. albicans* adhesins implicated in biofilm formation and virulence. Clade II strains of *C. auris*, which predominantly cause ear infections, have lost significant sections of subtelomeric regions that are enriched in putative adhesins, but are conserved in strains from Clades I, III, and IV (Muñoz et al., 2019). Perhaps future studies will unravel what role these subtelomeric-encoded adhesins have in human colonization and virulence. Recent studies have identified differentially regulated adhesin expression under physiologically-relevant conditions, including growth at 37°C in RPMI medium (Kean et al., 2018; Yue et al., 2018).

Several GPI-anchored cell wall proteins contribute to microbe-microbe and microbe-host adherence. *ALS4* is a member of the well-characterized *ALS* adhesin gene family in *C. albicans* (Zhao et al., 2005) and its ortholog is also differentially expressed during *C. auris* filamentous growth (Yue et al., 2018). Other GPI-anchored cell wall genes and potential adhesins were upregulated during *C. auris in vitro* biofilm formation compared to planktonic cells (*IFF4, CSA1, PGA26, PGA52*, and *HYR3*) (Kean et al., 2018). Furthermore, putative orthologs of *ALS3*, the *C. albicans* adhesin and invasin that induces host cell endocytosis of *C. albicans* hyphae (Liu and Filler, 2011), were detected on the *C. auris* cell surface by an anti-Als3p antibody (Singh et al., 2019). In addition, two *ALS* family orthologs, *ALS1* and *ALS5*, were expressed in biofilms (Kean et al., 2018). Each adhesin may play an important role in *C. auris* biofilm formation, persistence in hospital environments, and virulence, but further molecular studies are required to tease apart their independent contributions.

Several efflux pumps were induced in *C. auris* biofilms versus planktonic cells (Kean et al., 2018). ATP-binding cassette (ABC) transporters *SNQ2* and *CDR1* and major facilitator superfamily (MFS) genes *YHD3, RDC3*, and *MDR1* were upregulated between 2- and 4-fold in mature 24 h biofilms compared to planktonic cells. In line with these observations, efflux pump activity was elevated in mature biofilms relative to planktonic cells (Kean et al., 2018). Altogether, these findings suggest that biofilm formation may promote *C. auris* multi-drug resistance and virulence. Therefore, as with *C. albicans*, clinical measures to tackle *C. auris* drug resistance should consider the possibility that biofilms may harbor more drug-resistant cells and reduce the bioavailability of therapeutics.

### Stress Resistance and Persistence

One of the fascinating traits linked to *C. auris*’ emergence as a nosocomial pathogen is its ability to persist on abiotic hospital surfaces under stringent cleaning protocols (Schelenz et al., 2016; Welsh et al., 2017). Viable *C. auris* can be recovered from plastic surfaces or be detected via esterase activity for up to 2 and 4 weeks post-contamination, respectively (Welsh et al., 2017). These findings pose an interesting question: can *C. auris* form viable but non-culturable cells that persist in hospital environments? Although no studies to date have directly addressed this question, current metabolomic, transcriptomic, and molecular studies hold clues to *C. auris’* stress tolerance and persistence.

*C. auris* metabolism favors respiration as evidenced by enrichment in glycolytic and sugar transporter gene expression during yeast growth (Yue et al., 2018) and TCA cycle protein enrichment compared to *C. albicans* (Zamith-Miranda et al., 2019). Respiratory metabolism enhances ATP production and can reduce oxidative stress in *C. albicans*, thus promoting *in vivo* fitness and fluconazole resistance (Guo et al., 2017). Furthermore, *C. auris* lipid profiles show elevated levels of ergosterol and structural lipids compared to *C. albicans*, which may influence stress and antifungal resistance (Zamith-Miranda et al., 2019).

In addition, iron transport (Kean et al., 2018) and iron metabolism-associated genes (Yue et al., 2018) were upregulated in *C. auris* biofilm and filamentous cells, respectively. The *C. auris* genome provides evidence for expansion of siderophore-based iron transporters compared to *C. albicans*, further highlighting the importance of iron acquisition in this pathogen (Muñoz et al., 2018). Iron is an essential micronutrient that is depleted *via* nutritional immunity mechanisms *in vivo*, thus upregulation of iron uptake and metabolism-associated genes may improve *C. auris* fitness during infection. However, the contribution of *C. auris* iron transport gene families to virulence and drug resistance are currently unknown. Still, the expansion of the iron transport gene family suggests that iron deprivation may play an influential role in *C. auris*’ environmental reservoir.

Recent evidence has demonstrated that the stress-activated protein kinase Hog1 plays an evolutionarily conserved role in *C. auris* stress resistance, cell wall homeostasis, and virulence (Day et al., 2018). *C. auris* Hog1 shares 87% identity with the *C. albicans* Hog1 sequence. Consistent with findings from other *Candida* species, *C. auris HOG1* was required for osmotic stress resistance and SDS protection. However, *C. auris HOG1* was not required for weak acid or nitrosative stress tolerance, which contrasts its role in *C. glabrata* and *C. albicans*, respectively (Day et al., 2018). However, we do not know which *C. auris* genes are regulated in a Hog1-dependent manner or which Hog1 targets are responsible for these stress-adaptive phenotypes. Future transcriptomics and molecular studies will hopefully reveal which *C. auris* stress response genes are regulated via Hog1, which will highlight the evolutionary diversification of stress response circuitry across *Candida* species.

## Epigenetics and Gene Regulation in *C. auris*

Phenotypic plasticity is the interaction between a given genotype and the environment to produce multiple phenotypes. This plasticity is often the result of alterations in gene expression, which can be influenced by genetic or epigenetic mechanisms. In pathogens, phenotypic plasticity is often viewed as system for evolutionary bet-hedging to ensure microbial survival under shifting and/or hostile environmental conditions (Carja and Plotkin, 2017).

In the case of *C. auris*, very little is known about epigenetics and gene regulation or how these contribute to pathogenesis. As with other biological and virulence traits, early insights into *C. auris* epigenetics have been informed by previous work in *C. albicans*. Chromatin modification, including histone acetylation and deacetylation, plays an important role in *C. albicans* virulence (Garnaud et al., 2016; Kuchler et al., 2016). *C. auris* histone proteins and modifying enzymes were differentially expressed during filamentous growth relative to yeast, suggesting that epigenetic mechanisms may influence gene expression patterns in yeast and FC cells (Yue et al., 2018). In fact, the observation that *in vivo* passage can trigger a heritable, filamentation-competent phenotype is suggestive of epigenetic regulation, but more investigation is required to understand the mechanisms underlying this trait and its role in virulence.

Currently, available data demonstrates that *C. auris* has significant similarities in gene expression for important established virulence mechanisms in *C. albicans*, but there are also significant differences (Kean et al., 2018; Yue et al., 2018). Phenotypic heterogeneity between different *C. auris* clades provides an opportunity to understand how genetic mechanisms contribute to pathogenesis (*via* for example gene knock out or mutagenesis studies). Indeed, the adaptation of *C. albicans* gene deletion technologies for use in *C. auris* will enable future molecular investigations to understand gene regulatory networks and virulence in this emerging pathogen. Consequently, the *C. albicans NAT1*-Clox system has been successfully employed to disrupt *C. auris HOG1* and demonstrate its conserved role in stress responses (Shahana et al., 2014; Day et al., 2018). Future pathfinding molecular studies should reveal the conservation and divergence of gene regulatory networks in *C. albicans* and *C. auris* and highlight new avenues for antifungal development.

## Summary

Genomics and other omics studies have provided significant insight into the biology and pathogenesis of *C. auris* since its emergence as a modern human fungal pathogen. However, several questions remain about the evolution and fitness of this multi-drug resistant yeast which require further study.

## Author Contributions

All authors wrote and reviewed the manuscript and contributed and worked on figures.

## Conflict of Interest

The authors declare that the research was conducted in the absence of any commercial or financial relationships that could be construed as a potential conflict of interest.
